# Microbial genome sequencing

**DOI:** 10.1038/35021244

**Published:** 2000-08-17

**Authors:** Claire M. Fraser, Jonathan A. Eisen, Steven L. Salzberg

**Affiliations:** The Institute for Genomic Research, 9712 Medical Center Drive, Rockville, 20850 Maryland USA

## Abstract

Complete genome sequences of 30 microbial species have been determined during the past five years, and work in progress indicates that the complete sequences of more than 100 further microbial species will be available in the next two to four years. These results have revealed a tremendous amount of information on the physiology and evolution of microbial species, and should provide novel approaches to the diagnosis and treatment of infectious disease.

## Main

Microbes were the first organisms on Earth and preceded animals and plants by more than 3 billion years. They are the foundation of the biosphere, from both an evolutionary and an environmental perspective^1^. It has been estimated that microbial species comprise about 60% of the Earth's biomass. The genetic, metabolic and physiological diversity of microbial species is far greater than that found in plants and animals. But the diversity of the microbial world is largely unknown, with less than one-half of 1% of the estimated 2–3 billion microbial species identified. Of those species that have been described, their biological diversity is extraordinary, having adapted to grow under extremes of temperature, pH, salt concentration and oxygen levels. 

Perhaps no other area of research has been so energized by the application of genomic technology than the microbial field. It was only five years ago that The Institute for Genomic Research (TIGR) published the first complete genome sequence for a free-living organism, *Haemophilus influenzae*^2^; since that first report another 27 microbial genome sequences have been published, with at least 10–20 other projects at or near completion (for details see http://www.tigr.org/tdb/mdb/mdb.html). This progress represents, on average, one completed genome sequence every two months and all indications are that this pace will continue to accelerate. Included in the first completed microbial projects are many important human pathogens, the simplest known free-living organism, ‘model’ organisms, *Escherichia coli* and *Bacillus subtilis*, thermophilic bacterial species that might represent some of the deepest-branching members of the bacterial lineage, five representatives of the archaeal domain, and the first eukaryote, *Saccharomyces cerevisiae*. All of the organisms that have been studied by whole-genome analysis are species that can be grown either in the laboratory or in animal cells. It is important to remember that the vast majority of microbial species cannot be cultivated at all, and these organisms, which live in microbial communities, are essential to the overall ecology of the planet. Nevertheless, the study of ‘laboratory-adapted’ microbes has had a profound impact on our understanding of the biology and the evolutionary relationships between microbial species.

## Methods for whole-genome analysis

**Figure 1:**
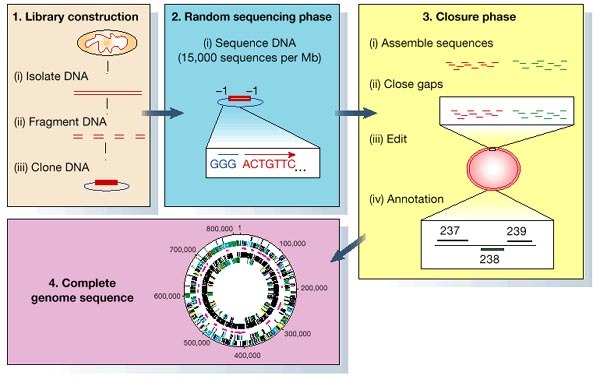
Diagram depicting the steps in a whole-genome shotgun sequencing project.

The method that was successfully used to determine the complete genome sequence of *H. influenzae* is a shotgun sequencing strategy ( Fig. 1). Before 1995, the largest genome sequenced with a random strategy was that of bacteriophage lambda with a genome size of 48,502 base pairs (bp), completed by Sanger *et al*. in 1982 (ref. 3). Despite advances in DNA-sequencing technology, the sequencing of whole genomes had not progressed beyond lambda-sized clones (about 40 kbp) because of the lack of sufficient computational approaches that would enable the efficient assembly of a large number of independent random sequences into a single contig.

For the *H. influenzae* and subsequent projects, we have used a computational method that was developed to create assemblies from hundreds of thousands of complementary DNA sequences 300–500-bp long^4^. This approach has proved to be a cost-effective and efficient approach to sequencing megabase-sized segments of genomic DNA. This strategy does not require an ordered set of cosmids or other subclones, thus significantly reducing the overall cost per base pair of producing a finished sequence, while providing high redundancy for accuracy and minimizing the effort required to obtain the whole genome sequence. The availability of improved technologies for longer sequence lengths (more than 700 bp) reduces problems associated with repetitive elements in the final assembly.

## Microbial gene finding and annotation

The identification of genes in prokaryotic genomes has advanced to the stage at which nearly all protein-coding regions can be identified with confidence. Computational gene finders using Markov modelling techniques now routinely find more than 99% of protein-coding regions^5^ and RNA genes^6^. Once the protein-coding genes have been located, the most challenging problem is to determine their function. Typically, about 40–60% of the genes in a newly sequenced bacterial genome display a detectable sequence similarity to protein sequences whose function is at least tentatively known. This sequence similarity is the primary basis for assigning function to new proteins, but the transfer of functional assignments is fraught with difficulties. 

To illustrate this problem, Table 1 contains an example showing the best matches in the database for a 1,344-bp gene from *Mycobacterium tuberculosis* at the time that the genome was being sequenced. All six of the best matches are kinases, but the specific names differ. A conservative naming strategy might use a family name that includes all six matches. Another strategy might use curated protein families (if they exist) to assign names; for example, the FGGY family named in the fourth line of Table 1 comes from the Pfam database^7^, a set of 1,815 hidden Markov models based on multiple alignments. By a closer examination of the literature, one could determine which of these protein names were based on laboratory experiments and which on sequence similarity. In any case, the assignment of a function to this protein requires the expertise of a skilled biologist. The rapidly changing nature of genome databases means that database searches must be repeated regularly to keep annotation accurate and up to date.

**Table 1:**
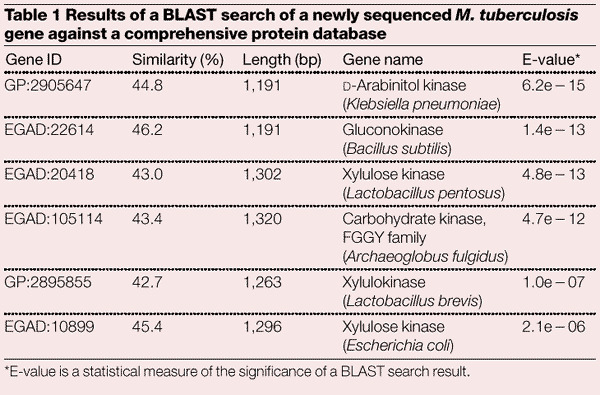
Results of a BLAST search of a newly sequenced *M. tuberculosis* gene against a comprehensive protein database

One possible solution to the annotation problem is to bring more of the resources of the scientific community to bear on each genome. No single centre can annotate all the functions of a living organism; experts from many different areas of biology should be encouraged to contribute to the annotation process. One possible model would be for geographically separated experts to deposit annotation to a central repository, which might also take on a curatorial or editorial role. An alternative model is one in which annotation resides in many different locations (as it does today), but in which new electronic links are created that allow scientists to locate rapidly all the information about a gene, genome or function. This latter model scales more easily and avoids the problem of overdependence on a single source.

## What have we learned from genome analysis?

Comparison of the results from 24 completed prokaryotic genome sequences, containing more than 50 Mbp of DNA sequence and 54,000 predicted open reading frames (ORFs), has revealed that gene density in the microbes is consistent across many species, with about one gene per kilobase (Table 2). Almost half of the ORFs in each species are of unknown biological function. When the function of this large subset of genes begins to be explained, it is likely that entirely novel biochemical pathways will be identified that might be relevant to medicine and biotechnology. Perhaps even more unexpected is the observation that about a quarter of the ORFs in each species studied so far are unique, with no significant sequence similarity to any other available protein sequence. Although this might at present be an artefact of the small number of microbial species studied by whole-genome analysis, it nevertheless supports the idea that there is tremendous biological diversity between microorganisms. Taken together, these data indicate that much of microbial biology has yet to be understood and suggest that the idea of a ‘model’ organism in the microbial world might not be appropriate, given the vast differences between even related species.

**Table 2:**
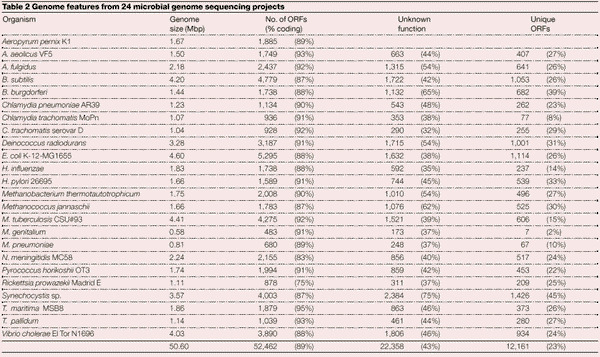
Genome features from 24 microbial genome sequencing projects

Our molecular picture of evolution for the past 20 years has been dominated by the small-subunit ribosomal RNA phylogentic tree that proposes three non-overlapping groups of living organisms: the bacteria, the archaea and the eukaryotes^8^. Although the archaea possess bacterial cell structures, it has been suggested that they share a common ancestor exclusive of bacteria.

Analysis of complete genome sequences is beginning to provide great insight into many questions about the evolution of microbes. One such area has encompassed the occurrence of genetic exchanges between different evolutionary lineages, a phenomenon known as horizontal, or lateral, gene transfer. The occurrence of horizontal gene transfer, such as that involving genes from organellar genomes to the nucleus, or of antibiotic resistance genes between bacterial species, has been well established for many years (see, for example, ref. 9). This phenomenon causes problems for studying the evolution of species because it means that some species are chimaeric, with different histories for different genes. Before the availability of complete genome sequences, studies of horizontal gene transfer had been limited because of the incompleteness of the data sets being analysed. Analyses of complete genome sequences have led to many recent suggestions that the extent of horizontal gene exchange is much greater than was previously realized^10,11,12^. For example, an analysis of the genomes of two thermophilic bacterial species, *Aquifex aeolicus* and *Thermotoga maritima*, revealed that 20–25% of the genes in these species were more similar to genes from archaea than those from bacteria^13,14^. This led to the suggestion of possible extensive gene exchanges between these species and archaeal lineages. But before one jumps to this conclusion it is important to consider the difficulties in inferring the occurrence of gene transfer. For example, the high percentage of genes with best matches to archaea in *A. aeolicus* and *T. maritima * could also be due to a high rate of evolution in the mesophilic bacteria (which would cause thermophilic and archaeal genes to have high levels of similarity despite their not having a common ancestry) or the loss of these genes from mesophilic bacteria^15^. For *T. maritima*, many lines of additional evidence support the assertion of gene transfer, including the observation that many of the archaeal-like genes occur in clusters in the genome, are in regions of unusual nucleotide composition, and branch in phylogenetic trees most closely to archaeal genes^14^. Most of the lines of evidence leading to assertions of horizontal gene transfer can have other causes. For example, unusual nucleotide composition can also arise from selection^16^, and differences in phylogenetic trees can be caused by convergence, inaccurate alignments^17^, long-branch attraction^18^ or sampling of different species^19^. It is therefore important to assess the evidence carefully and to find multiple types of evidence. This has yet to be done systematically, so we believe that it is too early to assign quantitative values to the extent of gene exchange between species.

Despite the apparent occurrence of extensive gene transfers in the history of microbes, it does seem that there might be a ‘core’ to each evolutionary lineage that retains some phylogenetic signal. The best evidence for this comes from the construction of ‘whole genome trees’ based on the presence and absence of particular homologues or orthologues in different complete genomes^20^. It is important to note that gene-content trees are averages of patterns produced by phylogeny, gene duplication and loss, and horizontal transfer; they are therefore not real phylogenetic trees. Nevertheless, the fact that these trees are very similar to phylogenetic trees of genes such as ribosomal RNA and RecA suggests that although horizontal gene transfer might be extensive, it is somehow constrained by phylogenetic relationships. Other evidence for a ‘core’ of particular lineages comes from the finding of a conserved core of euryarchaeal genomes^21,22^ and another finding that some types of gene might be more prone to gene transfer than others^23^. It therefore seems likely that horizontal gene transfer has not completely obliterated the phylogenetic signal in microbial genomes. Careful studies in which the phylogenetic trees of some of these core genes are compared across all genomes need to be done to see whether or not the core has a consistent phylogeny. Initial studies suggest that it does, at least for the major microbial groups^14^.

Although our ability to resolve patterns of the relationships among microbes is still limited, analysis of the genomes of closely related species is revealing much about genome evolution^24,25^. For example, a comparison of the genomes of four chlamydial species has revealed the occurrence of frequent tandem gene duplication and gene loss, as well as large chromosomal inversions^25^. Comparisons of closely related species should also reveal much about mutation processes, codon usage and other features that evolve rapidly^16^.

## Design of new antimicrobial agents and vaccines

One of the expected benefits of genome analysis of pathogenic bacteria is in the area of human health, particularly in the design of more rapid diagnostic reagents and the development of new vaccines and antimicrobial agents. These goals have become more urgent with the continuing spread of antibiotic resistance in important human pathogens. Moreover, results from the whole-genome analysis of human pathogens has suggested that there are mechanisms for generating antigenic variation in proteins expressed on the cell surface that are encoded within the genomes of these organisms. These mechanisms include the following: (1) slipped-strand mispairing within DNA sequence repeats found in 5′-intergenic regions and coding sequences as described for *H. influenzae*^2^, *Helicobacter pylori*^26^ and *M. tuberculosis*^27^, (2) recombination between homologous genes encoding outer-surface proteins as described for *Mycoplasma genitalium*^28^, *Mycoplasma pneumoniae*^29^ and *Treponema pallidum*^30^, and (3) clonal variability in surface-expressed proteins as described for *Plasmodium falciparum*^31^ and possibly *Borrelia burgdorferi *^32^. Experimental evidence from studies of clinical isolates of some species has demonstrated phenotypic variation in the relevant cell-surface proteins^33^, suggesting that, at least for human pathogens, the evolution of antigenic proteins probably occurs in real time, as cell populations divide. The ability of human pathogens to alter their antigenic potential and thereby evade the immune system has the potential to hinder vaccine development by conventional methods.

**Figure 2:**
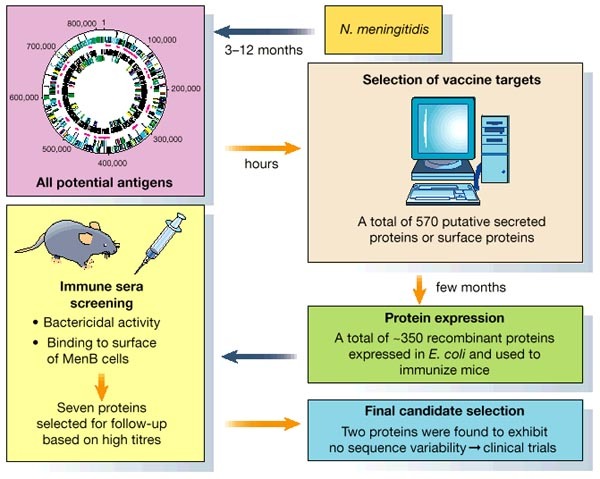
Diagram depicting how complete microbial genome sequence data can accelerate vaccine development.

Progress during the past year has supported the idea that complete genome sequence information can be exploited in the design of new vaccines and antimicrobial compounds. As an example, the identification of new vaccine candidates against serogroup B *Neisseria meningitidis* (MenB) was reported by Pizza *et al*. using a genomics-based approach^34^ ( Fig. 2). With the use of the entire genome sequence of a virulent serogroup B strain^35^, 570 putative cell-surface-expressed or secreted proteins were identified; the corresponding DNA sequences were cloned and expressed in *E. coli*. Of the putative targets, 61% were expressed successfully and used to immunize mice. Immune sera were screened for bactericidal activity and for the ability to bind to the surface of MenB cells. Seven representative proteins were selected for further study and were evaluated for their degree of sequence variability among multiple isolates and serogroups of *N. meningitidis *. Two highly conserved vaccine candidates emerged from this large-scale screening effort, which occurred in parallel with the completion of the genome sequence of *N. meningitidis*. These results provide the first definitive demonstration of the potential of genomic information to expand and accelerate the development of vaccines against pathogenic organisms.

Another example illustrates the potential of genomics to accelerate the development of novel antimicrobial agents. Jomaa *et al*.^36^ identified two genes in *P. falciparum* from sequence data from the malaria genome consortium that encode key enzymes in the 1-deoxy-d-xylulose-5-phosphate (DOXP) pathway that are required for the synthesis of isoprenoids such as cholesterol^37^. The DOXP pathway functions in some bacteria, algae and higher plants to produce isopentenyl diphosphate, a precursor of isoprenoids. In *P. falciparum*, the enzymes of the DOXP pathway are probably associated with a specialized organelle derived from algae called the apicoplast; they are expressed when the parasite is growing within red blood cells. Inhibitors of one of the key enzymes in the DOXP pathway, DOXP reductoisomerase, had previously been identified and had been shown to inhibit the bacterial enzyme and the growth of some bacterial species. Jomaa *et al*.^36^ demonstrated that two inhibitors of DOXP reductoisomerase, fosidomycin and FR900098, were able to inhibit the growth of *P. falciparum in vitro* and cure mice infected with a related species of *Plasmodium *. Both of these compounds exhibit low toxicity and high stability and are relatively inexpensive to produce, suggesting that they might be the basis of a potentially important new class of anti-malarial drugs.

## Conclusions

So far, studies in genomics have only scratched the surface of microbial diversity and have revealed how little is known about microbial species. In the next few years, more than 100 projects for sequencing microbial genomes should be completed, providing the scientific community with information on more than 300,000 predicted genes. A significant number of these genes will be novel and of unknown function. These novel genes represent exciting new opportunities for future research and potential sources of biological resources to be explored and exploited. The benefits of comparative genomics in understanding biochemical diversity, virulence and pathogenesis, and the evolution of species has been unequivocally demonstrated and the usefulness of comparative techniques will improve as more genomes become available. One of the major challenges is to develop techniques for assessing the function of novel genes on a large scale and integrating information on how genes and proteins interact at the cellular level to create and maintain a living organism. It is not unreasonable to expect that, by expanding our understanding of microbial biology and biodiversity, great strides can be made in the diagnosis and treatment of infectious diseases and in the identification of useful functions in the microbial world that could be applied to agricultural and industrial processes.
